# Multiscale superpixel depth feature extraction for hyperspectral image classification

**DOI:** 10.1038/s41598-025-90228-4

**Published:** 2025-04-19

**Authors:** Qi Yan, Shuzhen Zhang, Xiang Chen, Ziyou Zheng

**Affiliations:** College of Communication and Electronic Engineering, Jishou University, People’s South Road, Jishou, 416000 Hunan China

**Keywords:** Multiscale superpixel, Hyperspectral image classification, Statistical feature, Adaptive fusion strategy, Computational science, Computer science, Software, Applied optics, Optical techniques

## Abstract

Recently, superpixel segmentation has been widely employed in hyperspectral image (HSI) classification of remote sensing. However, the structures of land-covers in HSI commonly vary greatly, which makes it difficult to fully fit the boundaries of land-covers by single-scale superpixel segmentation. Moreover, the shape-irregularity of superpixel brings challenge for depth feature extraction. To overcome these issues, a multiscale superpixel depth feature extraction (MSDFE) method is proposed for HSI classification in this article, which effectively explores and integrates the spatial-spectral information of land-covers by adopting multiscale superpixel segmentation, constructing statistical features of superpixel, and conducting depth feature extraction. Specifically, to exploit rich spatial information of HSI, multiscale superpixel segmentation is firstly applied on the HSI. Once superpixels on different scales are obtained, two-dimensional statistical features with a united form are constructed for these superpixels with different spatial shapes. Based on these two-dimensional statistical features, a convolutional neural network is utilized to learn deeper features and classify these depth features. Finally, an adaptive strategy is adopted to fuse the multiscale classification results. Experiments on three real hyperspectral datasets indicate the superiority of the proposed MSDFE method over several state-of-the-art methods.

## Introduction

Hyperspectral images (HSI) contain spectral information with hundreds of continuous bands and two-dimensional spatial information, which can be widely applied in various fields, such as military reconnaissance, environmental monitoring, and precision agriculture^1–5^. In many remote sensing applications, it is necessary to classify each pixel in HSI^6,7^. However, the high-dimensional characteristics of HSI may lead to the Hughes phenomenon^8^, which decreases the classification performance. Furthermore, the complex spatial-spectral characteristics are difficult to articulate^9,10^. To alleviate these problems, various feature extraction methods for HSI have been explored.

Recently, deep learning models have shown great potential in the HSI classification^11^. Deep learning methods utilize multi-layer neural networks to extract abstract semantic features from data, which emulate the structure and function of the human brain’s neural networks. For example, recurrent neural network (RNN) is proposed to learn discriminative features by considering the spectral feature as sequential data^12^. Hang et al.^13^ propose a cascaded RNN model utilizing gated recurrent units, which is further extended into a spectral-spatial joint model by incorporating convolutional layers. Liu et al.^14^ first explore the usefulness and effectiveness of a generative adversarial network (GAN) for HSI classification. In various deep learning models, convolutional neural networks (CNNs) are extensively deployed in HSI classification. The CNNs execute convolutional operations on hyperspectral data framed within rectangular windows, which can extract profound semantic features combining spatial and spectral characteristics^15^. For instance, Roy et al.^16^ propose a hybrid spectral CNN (HybridSN), where 3DCNN first performs joint spatial-spectral feature representation, and 2DCNN further captures spatial representations at higher abstraction levels. Yu et al.^17^ adopt a simplified 2D-3D CNN architecture for HSI classification. Specifically, the 2-D convolutional layer aims to extract the spatial features encapsulated spectral information. The 3-D CNN approach focuses on harnessing band co-relation data. Lee et al.^18^ describe a contextual deep CNN, which forms joint spatial-spectral feature maps through multi-scale filters. Various CNN-based HSI feature extraction methods continue to emerge^19–21^, the above methods have demonstrated satisfactory classification performance, proving that CNN can improve the representation of spatial-spectral features. However, these methods still have a key problem. Most CNN methods extract spatial-spectral features through rectangular windows, it is difficult to characterize the irregularities of terrain boundaries. The limited ability of the rectangular window to describe boundary features may lead to misclassification of edge pixels.

In order to solve the problem of rectangular windows in effectively capturing boundary information, superpixel segmentation technology is considered. Superpixel segmentation adaptively divides adjacent pixels in natural images with similar characteristics such as color, brightness, and texture into non-overlapping sub-regions^22,23^. Each sub-region exhibits high internal pixel similarity, thereby preserving the spatial structural information of the image more effectively^24^. Utilizing superpixel segmentation for feature extraction in HSI is a promising approach. For instance, in^25^ and^26^, the segmented superpixels are combined with principal component analysis (PCA) for unsupervised feature extraction. Zhao et al.^27^ propose a superpixel-guided deformable convolution to make the shape of the deformable convolution align with the land coverage shape. Zhang et al.^28^ use superpixel-level hybrid discriminant analysis to exploit local/non-local spatial-spectral correlation information among/between superpixels for learning feature representations. However, the above-mentioned methods are based on single-scale superpixel segmentation. For the single-scale superpixel segmentation, it is a challenge to determine the optimal numbel of segmented superpixel. Furthermore, single-scale segmentation may result in over-segmentation or under-segmentation of some local areas, leaving the complex boundary information of certain land covers insufficiently captured. This limitation ultimately hinders the improvement in classification performance.

To overcome the limitations of single-scale superpixel segmentation, a multiscale approach is introduced to capture richer and more comprehensive boundary information. Multiscale superpixel segmentation methods can obtain richer feature information at different spatial scales, thereby improving the accuracy of classification algorithms. For example, in^29^ and^30^, multiscale superpixel-level data is used as a substitute for pixel-level data, where the average spectral vector of the superpixels is taken as their feature. Zhang et al.^31^ utilize multiscale superpixel-based sparse representation to acquire diverse spatial information through multiple scales of segmentation. Dundar et al.^32^ present multiscale superpixels and guided filters to get local information from different region scales. Wang et al.^33^ employ a multi-scale superpixel-guided structural profile method for HSI classification. Li et al.^34^ utilize a band-by-band adaptive multiscale superpixel feature extraction method to mitigate the difficulty of choosing the optimal superpixel scale, effectively harnessing available spectral and spatial information across bands. All these methods have been demonstrated to achieve satisfactory classification performance. However, the fusion of multi-scale information has the problem of assigning appropriate weights to each scale.

Based on the above comprehensive analysis, a novel multiscale superpixel depth feature extraction (MSDFE) method is proposed for HSI classification. Specifically, the superpixel segmentation method is applied to the dimensionality-reduced HSI to generate multi-scale 3D superpixel blocks. Then, two-dimensional statistical features, which are only determined by the spectral dimension, are constructed. After that, the statistical features of different scales are passed through a deep convolution module to extract deeper features. Finally, for each single-scale depth feature, the single-scale classification result of the HSI is obtained through a fully connected module, and an adaptive voting strategy is adopted to allocate weights and merge classification results from different scales. In this method, the statistical features effectively integrate the spatial-spectral information of the superpixel in the HSI. At the same time, the statistical features from different scales share the same size, which facilitates uniform input to the CNN model and performs deep feature extraction. Moreover, the adaptive fusion strategy comprehensively integrates multi-scale information, resulting in finer and more detailed predictions for HSI classification.

The rest of this article is structured as follows. The “Related works” Section briefly introduces the related works. In the “Proposed method” Section, the proposed MSDFE method is described in detail. In the “Experimental Results and Discussions” Section, the experimental results and analysis are provided. Finally, the conclusion is given in the “Conclusion” Section.

## Related works

### Simple linear iterative clustering

Simple linear iterative clustering (SLIC), proposed in 2010, adapts a K-means clustering approach. Despite its speed and simplicity, SLIC handles boundaries as well as or better than other segmentation methods^35,36^. It transforms color images into five-dimensional feature vectors composed of the CIELAB color space $[l\ a\ b]^T$ and the pixel’s position $[x\ y]^T.$ Then, it establishes a distance measurement standard and performs local clustering on the image pixels.

To create approximately similarly sized superpixels, the distance between the centers of the superpixels is set as:

1 $$\begin{aligned} S = \sqrt{\frac{N}{k}} \end{aligned}$$

where *N* is the number of pixels in the image and *k* represents the number of superpixels. Thus, it can be understood that the average area of the superpixel is *N*/*k*.

Subsequently, during the iteration process, the *i*th pixel is associated with the closest cluster center, where the search region of the cluster center overlaps the pixel’s location. Since the average spatial size of the superpixel is $S \times S,$ the cluster center searches for similar pixels in a $2S \times 2S$ region around it. In order to evaluate distance $d_s$ between the *i*th pixel and cluster center, the algorithm fuses the color proximity and spatial proximity into a single measure. $d_s$ is defined as:

2 $$\begin{aligned} d_{lab}&= \sqrt{(l_j-l_i)^2+(a_j-a_i)^2+(b_j-b_i)^2} \end{aligned}$$

3 $$\begin{aligned} d_{xy}&= \sqrt{(x_j-x_i)^2+(y_j-y_i)^2} \end{aligned}$$

4 $$\begin{aligned} d_s&= \sqrt{(d_{lab})^2+(\frac{d_{xy}}{S})^2m^2} \end{aligned}$$

where $d_{lab}$ is the color distance, $d_{xy}$ is the spatial distance, $d_s$ is the final distance metric used, $(l_j, a_j, b_j)$ and $(l_i, a_i, b_i)$ represent respectively the CIELAB color spaces of pixel *j* and the cluster center *i*, $(x_j, y_j)$ and $(x_i, y_i)$ are the spatial coordinates of pixel *j* and the cluster center *i*, respectively. The constant *m* is used to weigh the balance between color similarity and spatial proximity.

Once each pixel has been associated with the closest superpixel center, the average vector of all pixels belonging to the superpixel is calculated as the new cluster center. The Euclidean distance is then used to compute a residual error between the previous superpixel center and the new superpixel center. The process is repeated iteratively until the residual error falls below a predefined threshold.

### Covariance statistical feature

Covariance is a statistical measure used to quantify the linear relationship between two random variables. It describes how two variables vary together, reflecting the trend of their correlation. Covariance measures the degree of linear dependence between two variables, *X* and *Y*. The formula is expressed as follows:

5 $$\begin{aligned} Cov(X,Y) = \frac{1}{n}\sum _{i=1}^{n}(X_i-\bar{X})(Y_i-\bar{Y}) \end{aligned}$$

where $X_i$ and $Y_i$ represent the sample values of two variables, while $\bar{X}$ and $\bar{Y}$ denote the means of variables *X* and *Y*, respectively. *n* is the sample size. Covariance is an important metric for describing the linear relationship between two variables. It measures the trend of variation between the variables. The covariance statistical characteristic in the proposed method will be used to depict the correlation information between different bands of HSI.

## Proposed method

**Fig. 1:**
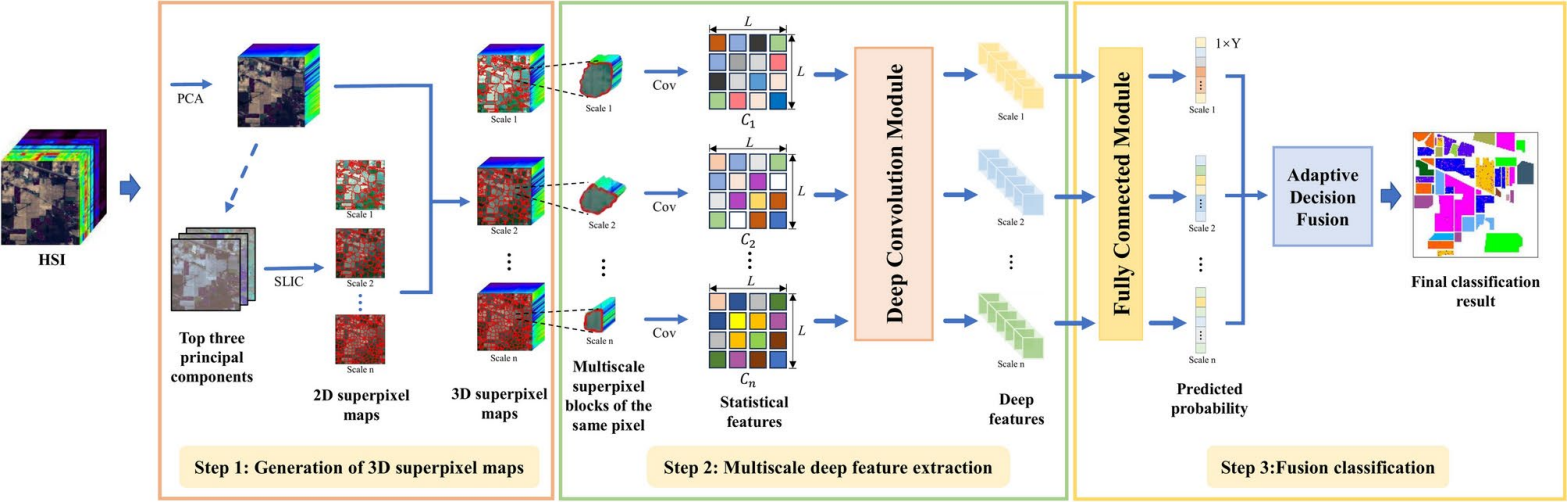
Flowchart of the proposed MSDFE method.

In this article, we propose a novel MSDFE method, which mainly consists of the following three parts: (1) generation of 3D superpixel maps; (2) multiscale deep feature extraction; and (3) fusion classification. The flowchart of the proposed MSDFE method is illustrated in Fig. 1 and the method details are provided as follows.

### Generation of 3D superpixel maps

In HSI, neighboring pixels always show similar spectral characteristics and high spatial correlation, it is effective to use superpixel segmentation to capture spectral similarity and spatial correlation between pixels. As shown in Fig. 1, to select more informative bands and reduce computational complexity, the PCA method is applied to the original HSI. Specifically, given an HSI defined by ${\textbf {Z}} \in \mathbb {R} ^{M \times H \times K},$ the dimension-reduced image ${\textbf {X}} \in \mathbb {R}^{M \times H \times L}$ can be obtained by the PCA method, where *M* and *H* are the size of the spatial dimensions, *K* is the number of original spectral bands, and *L* represents the number of PCA principal components ($L \ll K$). Then a 2D superpixel map for the first three principal components is gained by adopting the SLIC method, which consists of *m* irregular and non-overlapping superpixel regions. The 2D superpixel map is finally combined with the dimension-reduced HSI to generate a 3D image labeled by superpixels. To obtain multiscale structural information, multiscale segmentation with different superpixel numbers for the same HSI is applied, which generates multi-scale superpixel maps.

**Fig. 2:**
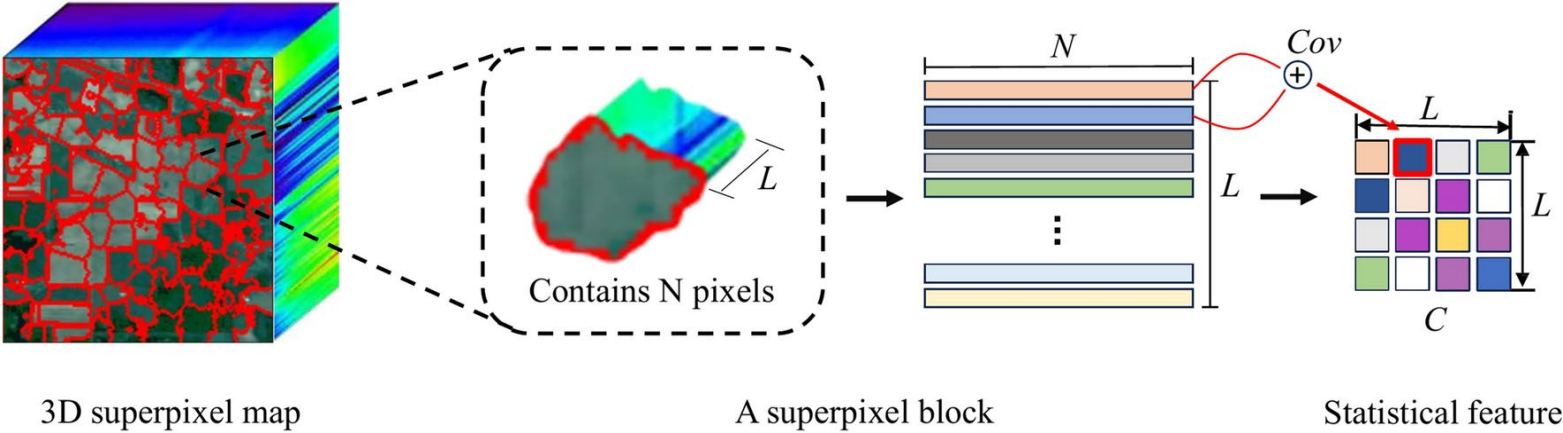
Illustration of the 2D statistical feature generation for a superpixel block.

### Multiscale deep feature extraction

For each pixel, a series of superpixel blocks with different shapes are obtained by multiscale superpixel segmentation. Although different superpixel blocks have different shapes, their statistical features usually share the same size, which simultaneous extracts the spatial and spectral information from the superpixel blocks. Moreover, the shallow feature expression in a unified form can be further input into the deep network to mine the corresponding deep features. Therefore, for multiscale superpixel blocks of the same pixel, two-dimensional statistical features are first constructed, and subsequently, these obtained two-dimensional statistical features are input into a deep convolution module to extract the multiscale deep features of the pixel. The illustration of the 2D statistical feature generation for a superpixel block is shown in Fig. 2, where *N* is the number of pixels contained in a superpixel block, and *L* represents the number of dimension-reduced bands. The two-dimensional statistical feature map, which is the covariance matrix of the superpixel block, is calculated by Eq. (6). It can be noted that the size of the obtained covariance matrix is determined only by the number of dimension-reduced bands *L*, which means that the covariance matrices coming from superpixel blocks with different spatial shapes share the same size. The formula for the covariance matrix on one scale is extracted as follows:

6 $$\begin{aligned} C = \frac{1}{N-1} \sum _{i=1}^{N} (x_i- \mu )(x_i - \mu )^T \in \mathbb {R}^{L \times L} \end{aligned}$$

where $x_i$ is the *i*th pixel within the superpixel block, and $\mu$ denotes the mean spectral feature of *N* pixels within a superpixel block. Moreover, assuming a total of *n* segmentation scales, $C_k (k=1,\cdots ,n)$ is used to denote the *k*th covariance maps, which are obtained with (6) from the computation of *k*th scale superpixel block containing the sample pixel.

**Fig. 3:**
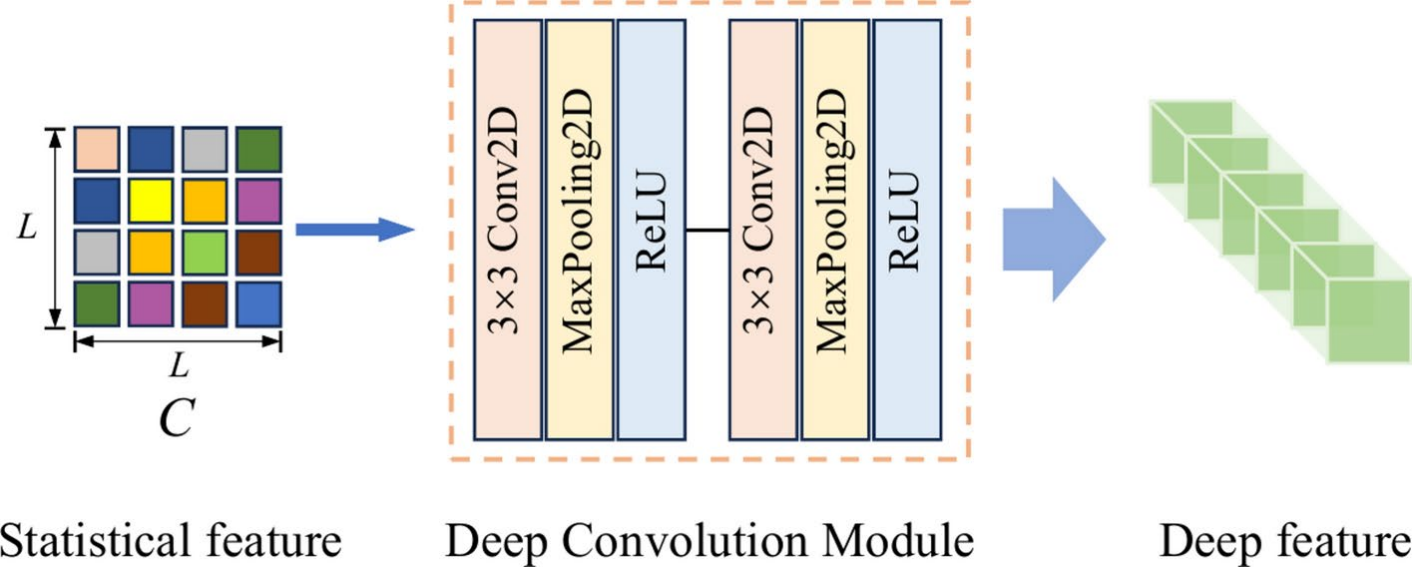
Illustration of the deep feature generation from the single-scale statistical feature.

**Table 1:** Layerwise parameter of the deep convolution module.

Layer type	Parameter
The first Conv2D	$128 \times 3 \times 3$,* stride = 1*
MaxPooling2D	$2 \times 2$,* stride = 2*
Activation	*ReLU*
The second Conv2D	$64 \times 3 \times 3$,* stride = 1*
MaxPooling2D	$2 \times 2$,* stride = 2*
Activation	*ReLU*

The obtained statistical feature, namely the covariance map, only represents the shallow feature of the superpixel block. Therefore, a deep network is considered to extract deeper features. As shown in Fig. 3, the statistical features are fed into a deep convolution module to extract deep features. The module consists of two convolutional layers followed by pooling layers. The ReLU activation functions are applied after each layer to introduce non-linearity. The module parameters are shown in Table 1. After the above process, multi-scale superpixel blocks of the same pixel obtain corresponding multi-scale depth features.

### Fusion classification

For each single-scale depth feature, the single-scale classification result of HSI is obtained through a fully connected module, which contains two fully connected layers consisting of a Dense layer and an activation function layer. In order to obtain the final classification result, we adopt an adaptive multi-scale fusion strategy. Most multiscale decision fusion methods nowadays use majority voting with uniform weights, which indicates that each scale has the same impact on the predicted results. By this approach, scales with poor classification performance may have an excessive influence on the fused classification result, which will reduce the final classification performance. In light of this, an adaptive decision fusion strategy is employed to assign different weights to different scales. The weight distribution rule^37^ is expressed as follows:

7 $$\begin{aligned} P&= \sum _{k=1}^n \lambda _k P_k(y = c \mid x) \in \mathbb {R}^{1 \times Y} \end{aligned}$$

8 $$\begin{aligned} \lambda _k&= \frac{X_k - X_{min}}{X_{max} - X_{min}} \end{aligned}$$

where *P* represents the predicted probability of sample *x* for each category, $P_k(y = c \mid x)$ represents the predicted probability at *k*th scale that sample *x* belongs to category *c*, *n* is the number of scales, and *Y* is the number of categories. The weight coefficients $\lambda _k$ of each scale are determined by Eq. (8). Finally, the category with the highest probability in *P* is chosen as the final prediction. $X_k$ is the overall classification accuracy (OA) of the *k*th scale, $X_{max}$ and $X_{min}$ represent the maximum and minimum values in the OA values after classification at all scales, respectively.

## Experimental results and discussions

### Datasets

To verify the performance of the proposed method, three real hyperspectral image datasets are used in the experiments: the Indian Pines dataset, the Salinas dataset, and the Pavia University dataset.

### Experimental setup

(1) *Indian Pines:* The Indian Pines dataset was gathered by AVIRIS sensor over the Indian Pines test site in northwestern Indiana, USA. The dataset consists of a spatial size of $145 \times 145$ pixels and 200 spectral reflectance bands after removing bands covering the region of water absorption. The wavelength ranges from 0.4 to 2.5 $\mu$m, and the spatial resolution is 20m per pixel. The dataset contains 16 categories, and detailed information about the dataset categories is shown in Table 2.

(2) *Salinas:* The Salinas dataset was collected by the AVIRIS sensor over Salinas Valley, California, USA. The dataset contains 204 spectral bands after discarding 20 water absorption bands. The spatial size is $512\times 217,$ and has the characteristic of high spatial resolution (3.7m per pixel). The dataset contains 16 categories, and detailed information about the dataset categories is shown in Table 2.

(3) *Pavia University:* The Pavia University dataset was captured by the ROSIS sensor during a flight campaign over the University of Pavia, Italy. The number of spectral bands in the dataset is 103, with $610\times 340$ pixels and a spatial resolution of 1.3 m. The dataset contains 9 categories, and detailed information about the dataset categories is shown in Table 2.

**Table 2:**
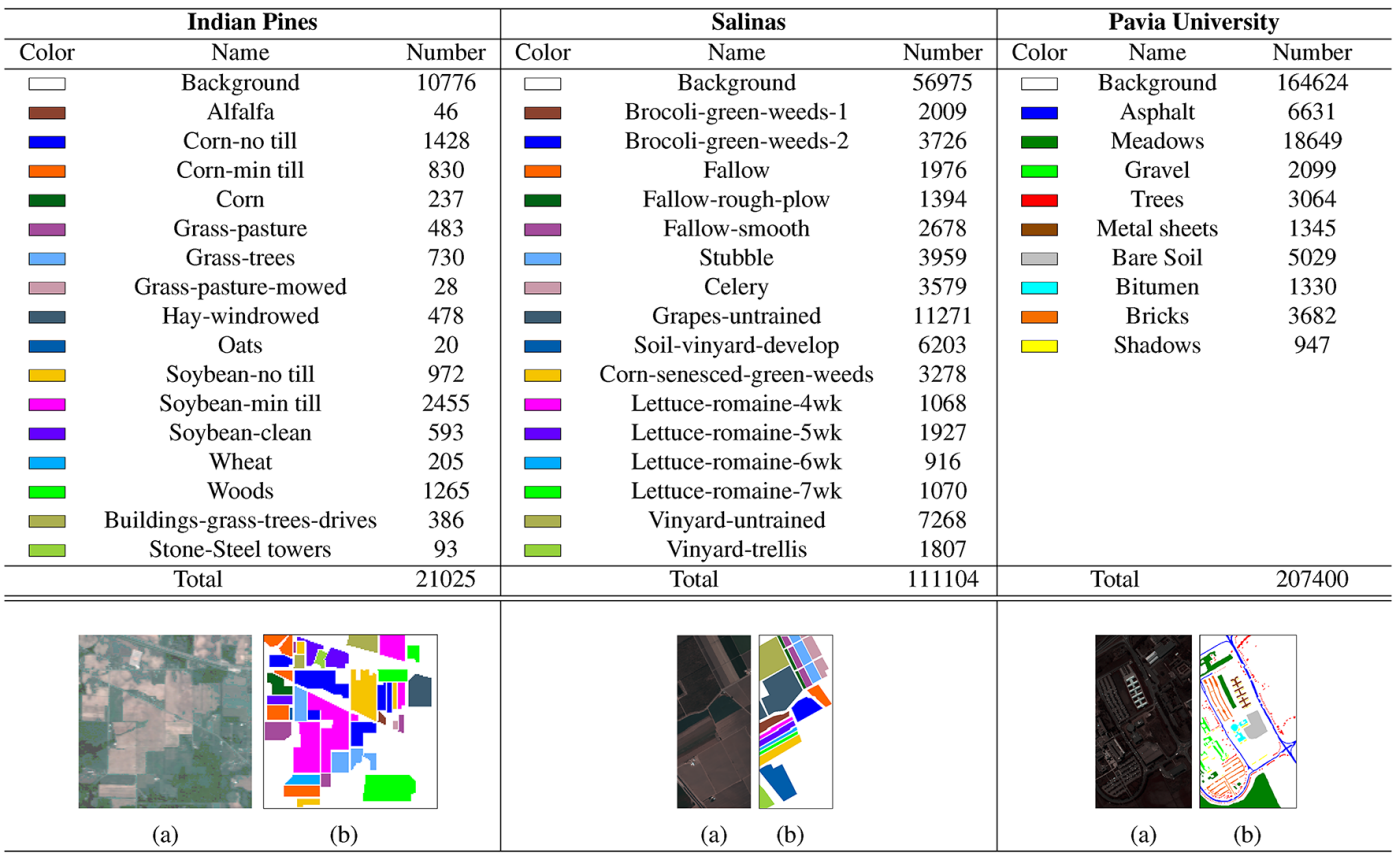
Number of Samples of The Test HSIs. (a) Pseudo-color map, (b) Ground truth.

To validate the performance of the proposed MSDFE method, various HSI classification methods, including 2D-CNN, multiscale covariance maps (MCMs)^7^, HybridSN^16^, spatial-spectral feature tokenization transformer (SSFTT)^38^, CNN-enhanced GCN (CEGCN)^39^, multilevel superpixel structured graph U-Nets (MSSGU)^40^, superpixel-based Brownian descriptor (SBD)^41^, superpixel-level hybrid discriminant analysis (SHDA)^28^, attention multi-hop graph and multi-scale convolutional fusion network (AMGCFN)^42^, are used to be compared. Considering that the proposed method is based on superpixel segmentation and utilizes CNN for deep feature extraction, the compared methods selected for the experiment focus on two aspects: feature learning respectively based on superpixel segmentation and CNN with fixed windows. The most of the compared methods (except for 2D-CNN, MCMs, HybridSN, and SFFTT) rely on superpixel. Among these methods, MCMs, MSSGU, and AMGCFN employ a multiscale strategy. In specific, 2D-CNN, MCMs, HybridSN, and SFFTT extract spectral-spatial features by utilizing the spectral-spatial information within a fixed square window neighborhood. In CEGCN, MSSGU, SBD, SHDA, and AMGCFN, the adaptive spatial structure information is obtained by employing the superpixel segmentation method to extract the spatial-spectral features. In CEGCN, the CNN and GCN branches are used to generate complementary spatial-spectral features for feature learning at the pixel and superpixel levels, respectively. In MSSGU, different-scale features are fused in a coarse-to-fine progressive manner to generate more subtle fused features for the pixelwise classification task. In SBD, the Brownian descriptor based on superpixels is used to extract linear and nonlinear spectral information. In SHDA, superpixels and discriminant analysis are integrated to learn feature representations.

For all the comparative algorithms, the corresponding public codes and consistent hyperparameters are employed to ensure that the comparative experiments are more convincing. The Xavier method is utilized to initialize all weights, while the biases are initialized to zero. The Adam optimizer is adopted for training. The learning rate is set to 0.001 and adaptively changes during the training process. The batch size is set to 100, and five samples per class are randomly selected as the training set. In addition, the experiments are conducted on a hardware environment composed of an i7-12400F CPU, 48 GB of RAM, and a graphics processing unit (GPU) NVIDIA GeForce RTX 4070 with 12 GB video memory. All experiments are repeated ten times, and four evaluation metrics, including the overall classification accuracy (OA), the average classification accuracy (AA), the kappa coefficient (*k*), and the classification accuracy per category, are introduced to analyze the effectiveness of these comparative methods. The higher the value of four metrics is, the better classification performance is.

**Fig. 4:**
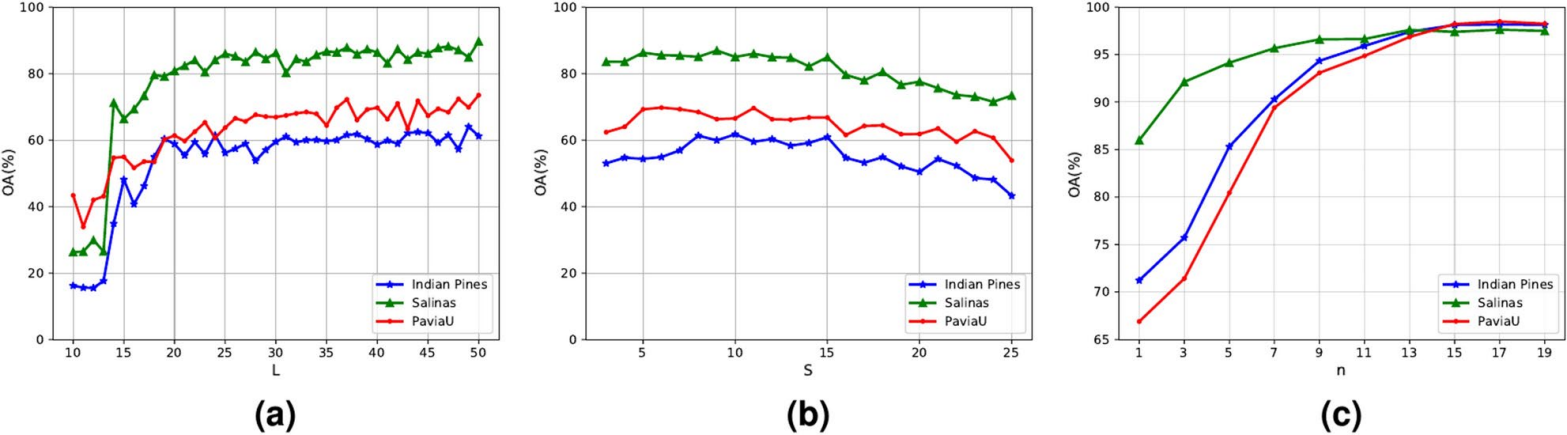
Parameter analysis experimental results of the proposed method. (**a**) Influence of the reduced dimensionality *L* on the classification performance of datasets under single-scale segmentation condition. (**b**) Influence of the parameter *S* related to the average superpixel area on the classification performance of datasets. (**c**) Influence of the number of superpixel scale *n* on classification performance for three datasets.

### Parameter analysis

In this section, a detailed explanation of the important parameters in the proposed MSDFE method is provided. For single-scale superpixel segmentation, the reduced dimensionality *L* and the parameter *S*, which is related to the average superpixel area (i.e., the basic superpixel center spacing *S* in Eq. (1)) will be discussed. For multiscale information fusion, the number of scales *n* will be analyzed in detail. When the influence of one parameter is analyzed, the other parameters are fixed to the default values.

Firstly, the influence of reduced dimensionality *L* on the classification results is analyzed. In the experiments, the parameter *L* is varied from 10 to 50 with step of 1. Figure 4a presents the connections of OA with the reduced dimensionality for the three datasets. With the increase in the number of dimensions *L*, the OA values first increase and then show a relatively stable trend around a certain value. For the Indian Pines, Salinas, and Pavia University datasets, the OA values tend to stabilize when *L* is greater than 22, 23, and 26, respectively. Considering that, when *L* is set to 30, compared methods exhibit relatively high and stable OA values for the three datasets. Therefore, in all comparative algorithms, the reduced dimensionality *L* is set to 30.

Then, the impact of the parameter *S* related to the average superpixel area, which reflects the distribution density of the superpixels, on the proposed MSDFE method is discussed. Larger spacing usually implies a smaller number of superpixels and a coarser segmentation, while smaller spacing indicates a larger number of superpixels and a more detailed segmentation. The impact of the OA values with the superpixel center spacing *S* for the three datasets is shown in Fig. 4b. In the experiments, the range of the parameter *S* is [3, 25], with a step size of 1. Theoretically, the resulting superpixel region becomes larger as *S* becomes larger. Too small superpixel may not effectively utilize the spatial information in one homogeneous region, while too large superpixel may contain some pixels from different classes in one superpixel. With the continuous increase of *S*, the OA values begin to rise, subsequently maintain relative stability, and finally continually decrease. When *S* is around 11, the OA values remain relatively stable for the three datasets. Hence, the basic superpixel center spacing is set to 11.

Finally, the effect of the number of the scale *n* on classification performance is evaluated. The different superpixel center spacings are obtained around the basic superpixel center spacing *S* with the step of 1 by adding and subtracting simultaneously. Figure 4c shows the correlation of the classification performance with the number of the scale for the three datasets. With the increase in the number of the scale *n*, the OA values initially increase continuously and then stabilize, when *n* reaches a certain level. This is mainly due to the fact that, with the increase of the number of the scale, the features of different classes can be captured more effectively and the spatial structure in HSI can be expressed comprehensively. However, an excessive number of scales may increase the redundancy of information within the samples, which could lead to the inability to extract more easily distinguishable features affecting classification. For the three datasets, the value of *n* for obtaining the best classification results is 15. Therefore, the number of the scale *n* is fixed to 15 for the proposed MSDFE method.

### Module ablation analysis

We conduct a module ablation analysis to verify the effectiveness of different components in the proposed method. Specifically, the experiments are divided into four groups: the 2DCNN with the composition of majority voting and the covariance features from rectangular windows of different sizes as a baseline method, the baseline method with the module of superpixel covariance maps (SCM), the baseline method with the module of adaptive decision fusion (ADF), and the baseline framework combining SCM and ADF (i.e., the proposed MSDF method).

**Table 3:** Classification accuracy (in percent) of the ablation experimental with five training samples per class

Methods	Indian Pines			Salinas			Pavia University		
	OA	AA	$\kappa$	OA	AA	$\kappa$	OA	AA	$\kappa$
Baseline	74.10	83.11	70.79	93.65	96.99	92.98	72.09	80.72	65.41
Baseline with SCM	93.63	95.35	92.75	94.46	96.70	93.86	91.20	93.02	91.02
Baseline with ADF	75.16	84.27	74.29	93.73	96.78	93.10	74.23	81.53	72.89
MSDEF	**98.12**	**98.96**	**97.86**	**97.60**	**99.27**	**97.34**	**98.23**	**98.79**	**97.65**

The best results are highlighted in bold

**Fig. 5:**
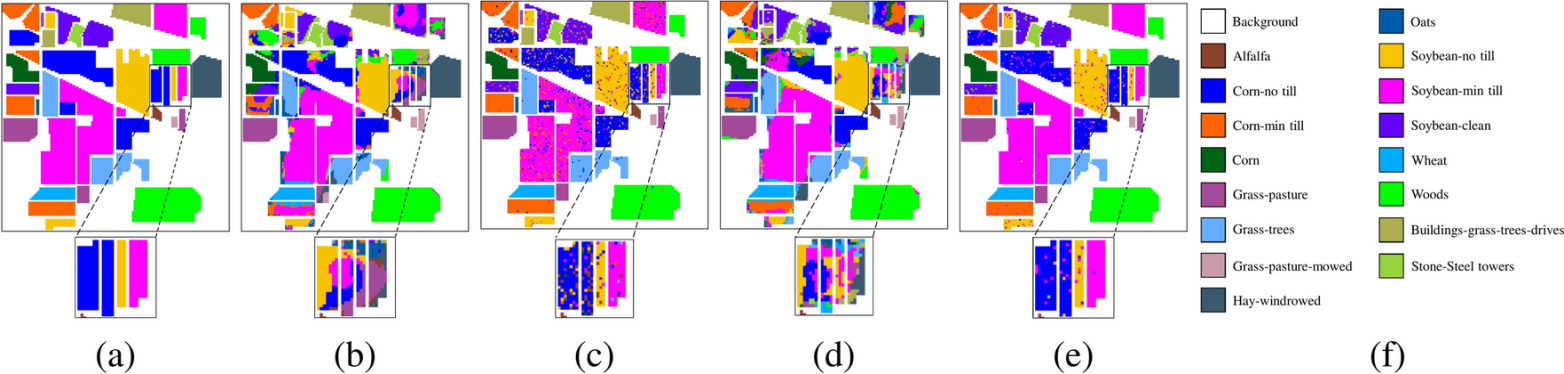
Ablation experimental results of the Indian Pines dataset. (**a**) Ground truth, (**b**) Baselines, OA = 75.13%, (**c**) Baseline+SCM, OA = 91.71%, (**d**) Baseline+ADF, OA = 73.21%, (**e**) MSDFE, OA = 97.85%, (**f**) Labels.

**Fig. 6:**
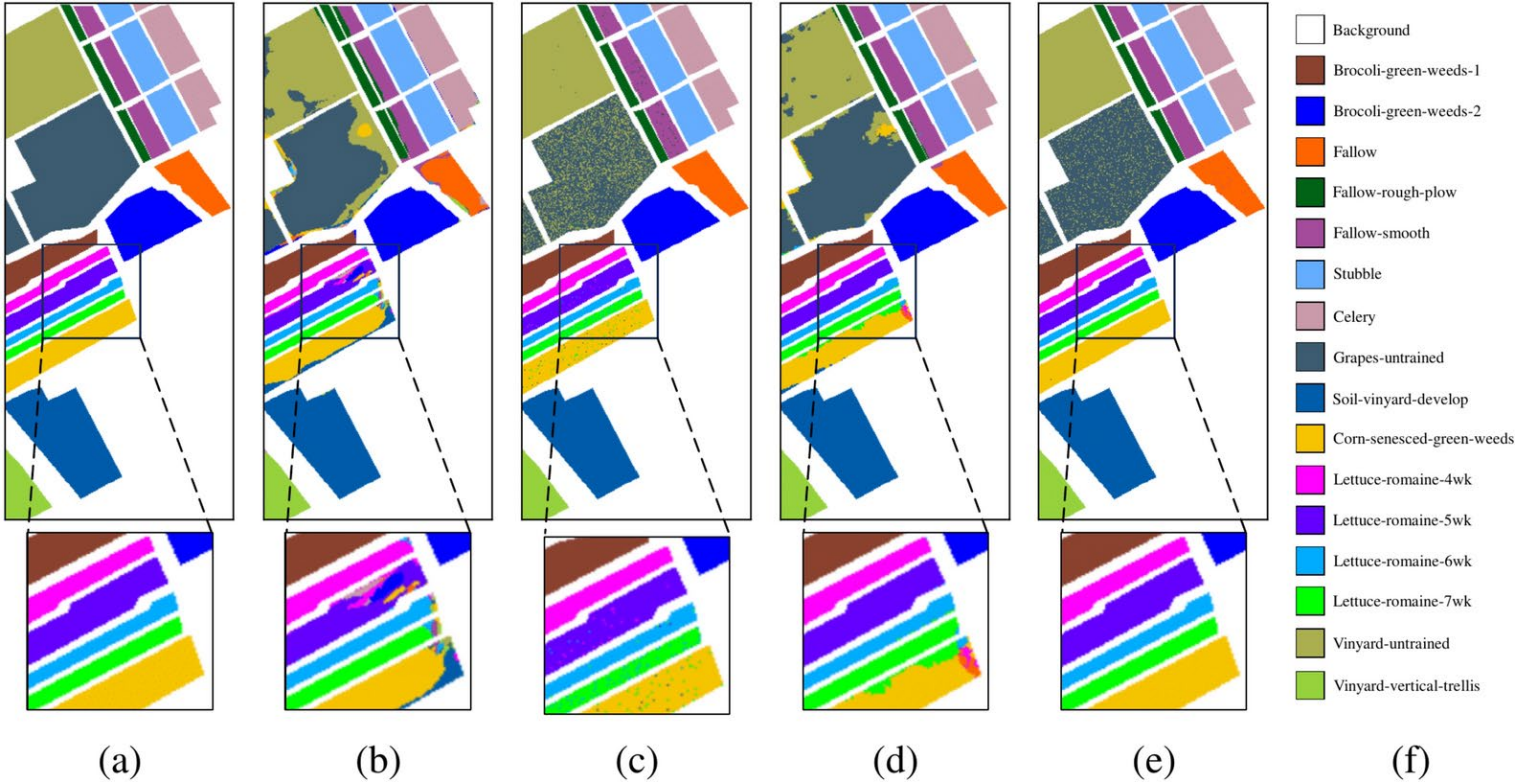
Ablation experimental results of the Salinas dataset. (**a**) Ground truth, (**b**) Baselines, OA = 90.32%, (**c**) Baseline+SCM, OA = 93.61%, (**d**) Baseline+ADF, OA = 93.03%, (**e**) MSDFE, OA = 97.85%, (**f**) Labels.

**Fig. 7:**
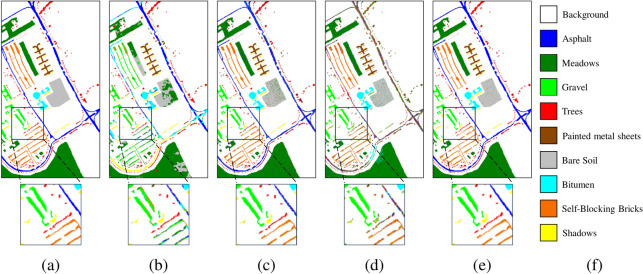
Ablation experimental results of the Pavia University dataset. (**a**) Ground truth, (**b**) Baselines, OA = 70.30%, (**c**) Baseline+SCM, OA = 91.44%, (**d**) Baseline+ADF, OA = 78.68%, (**e**) MSDFE, OA = 97.85%, (**f**) Labels.

The results of the ablation experiments are shown in Table 3 and the corresponding classification maps are provided in Figs. 5, 6, and 7. From the experimental results, it can be observed that the baseline method exhibits the poorest classification performance. Compared to the baseline, adding the ADF module improves classification accuracy by 1.06%, 0.08%, and 2.14%, respectively. This indicates that adaptive decision fusion effectively captures subtle differences across multiple scales and makes better use of multi-scale information. Additionally, the baseline method incorporating the SCM module achieves performance improvements of 19.53%, 0.81%, and 19.11%, respectively. It means that the superpixel covariance map effectively preserves the spatial-spectral information of HSI while capturing the boundary information of different land-cover types, significantly enhancing classification performance. To show the differences in the classification map more clearly, certain regions have been enlarged. It can be seen from the classification maps that the proposed method classifies the boundary pixels more accurately. For example, the classification map obtained by the proposed method on the Salinas data set is highly consistent with the ground truth. Therefore, it is further shown that the MSDFE method can effectively extract spatial information in HSI.

### Comparison with other methods

In this section, the proposed MSDFE method is compared with the classic and state-of-the-art classification methods to verify its effectiveness. The parameters of various comparative algorithms are set according to the related research articles or open-source codes. For the three datasets used in the experiments, five samples from each class are randomly selected as the training set, while the remaining samples are used as the testing set. The validation set is not split from the training or testing set (except for CEGCN, MSSGU, and AMGCFN). This means that the proportions of the training sets for the Indian Pines, Salinas, and Pavia University datasets are 0.78%, 0.15%, and 0.11%, respectively. A validation set is set up in CEGCN, MSSGU, and AMGCFN methods, which shares the same number of samples as the training set and is included in the test set.

**Table 4:** Classification accuracy (in percent) of different on Indian Pines dataset with five training samples per class

Class Names	2D-CNN	MCMs	HybridSN	SSFTT	CEGCN	MSSGU	SBD	SHDA	AMGCFN	MSDFE
Alfalfa	94.15(4.78)	93.04(5.03)	96.34(3.49)	99.27(1.56)	95.56(3.97)	99.27(1.12)	97.56(0.00)	97.56(0.00)	99.17(1.78)	**100.00(0.00)**
Corn-no till	31.48(13.53)	56.37(10.71)	31.01(9.83)	44.67(9.76)	42.91(12.42)	64.01(10.45)	64.17(11.05)	66.62(15.94)	55.18(9.47)	**92.29(3.37)**
Corn-min till	43.19(13.87)	50.36(6.00)	45.30(15.88)	56.32(8.38)	53.20(18.86)	59.06(17.35)	67.35(23.57)	86.12(12.27)	66.70(14.80)	**98.69(1.15)**
Corn	72.41(16.45)	99.32(1.15)	62.93(15.79)	88.84(10.24)	80.31(13.24)	94.31(6.04)	94.96(10.93)	85.00(17.02)	95.52(3.97)	**99.78(0.40)**
Grass-pasture	66.55(10.17)	68.61(1.18)	63.37(19.62)	83.22(6.00)	73.64(6.75)	75.50(10.85)	87.87(9.63)	80.96(10.27)	75.84(7.75)	**99.83(0.37)**
Grass-trees	81.97(7.63)	81.92(2.53)	85.24(10.91)	94.68(3.71)	97.94(0.89)	91.49(6.13)	95.41(4.36)	95.59(5.70)	86.74(5.64)	**99.66(0.37)**
Grass-pasture-mowed	99.56(1.30)	**100.00(0.00)**	**100.00(0.00)**	99.57(1.30)	**100.00(0.00)**	**100.00(0.00)**	96.09(1.37)	96.96(2.10)	**100.00(0.00)**	**100.00(0.00)**
Hay-windrowed	87.82(14.23)	**100.00(0.00)**	97.91(3.47)	92.22(11.30)	93.78(6.84)	93.91(7.96)	95.64(13.10)	96.96(8.96)	98.57(1.94)	99.98(0.06)
Oats	**100.00(0.00)**	**100.00(0.00)**	**100.00(0.00)**	**100.00(0.00)**	**100.00(0.00)**	**100.00(0.00)**	**100.00(0.00)**	**100.00(0.00)**	**100.00(0.00)**	**100.00(0.00)**
Soybean-no till	51.96(12.64)	71.11(5.04)	58.28(10.68)	70.53(11.91)	70.21(7.50)	73.86(8.26)	77.05(14.68)	72.81(15.70)	72.05(8.51)	**98.35(0.95)**
Soybean-min till	60.06(9.30)	75.33(2.00)	37.82(13.09)	49.93(11.99)	67.66(20.00)	74.35(17.43)	58.51(12.36)	79.02(10.65)	70.51(9.69)	**98.92(0.57)**
Soybean-clean	35.39(9.06)	56.49(7.77)	37.26(8.75)	53.13(10.85)	50.74(10.80)	70.00(12.16)	79.30(11.84)	82.67(10.60)	63.79(10.78)	**95.88(1.60)**
Wheat	99.75(0.51)	99.71(0.24)	96.80(4.88)	99.50(1.34)	99.69(0.47)	99.45(1.19)	99.50(0.00)	99.50(0.00)	97.28(3.33)	**100.00(0.00)**
Woods	80.70(9.12)	88.65(1.29)	67.69(17.38)	85.95(7.55)	92.02(8.52)	98.28(2.18)	91.92(8.07)	89.51(10.27)	85.81(19.94)	**100.00(0.00)**
Buildings-Grass-Trees-Drives	61.63(16.77)	88.86(3.48)	64.09(14.36)	83.18(13.73)	62.47(13.36)	88.27(9.71)	78.35(13.12)	84.09(11.35)	91.01(10.60)	**100.00(0.00)**
Stone-Steel-Towers	**100.00(0.00)**	**100.00(0.00)**	94.89(6.51)	**100.00(0.00)**	98.55(2.52)	98.75(2.00)	96.59(6.15)	**100.00(0.00)**	96.87(3.70)	**100.00(0.00)**
OA	59.96(3.10)	74.10(1.40)	54.14(4.64)	66.97(3.78)	70.11(3.87)	78.39(4.54)	75.88(3.93)	81.95(4.75)	74.66(3.63)	**98.12(0.51)**
AA	72.91(1.89)	83.11(0.62)	71.18(1.93)	81.31(2.03)	79.92(1.86)	86.28(1.80)	86.27(1.69)	88.34(3.38)	84.68(1.71)	**98.96(0.30)**
$\kappa$	54.94(3.24)	70.79(1.56)	49.54(4.44)	63.18(4.81)	66.00(4.08)	75.47(4.87)	72.94(4.28)	79.58(5.33)	71.48(3.98)	**97.86(0.58)**

The best results are highlighted in bold

**Fig. 8:**
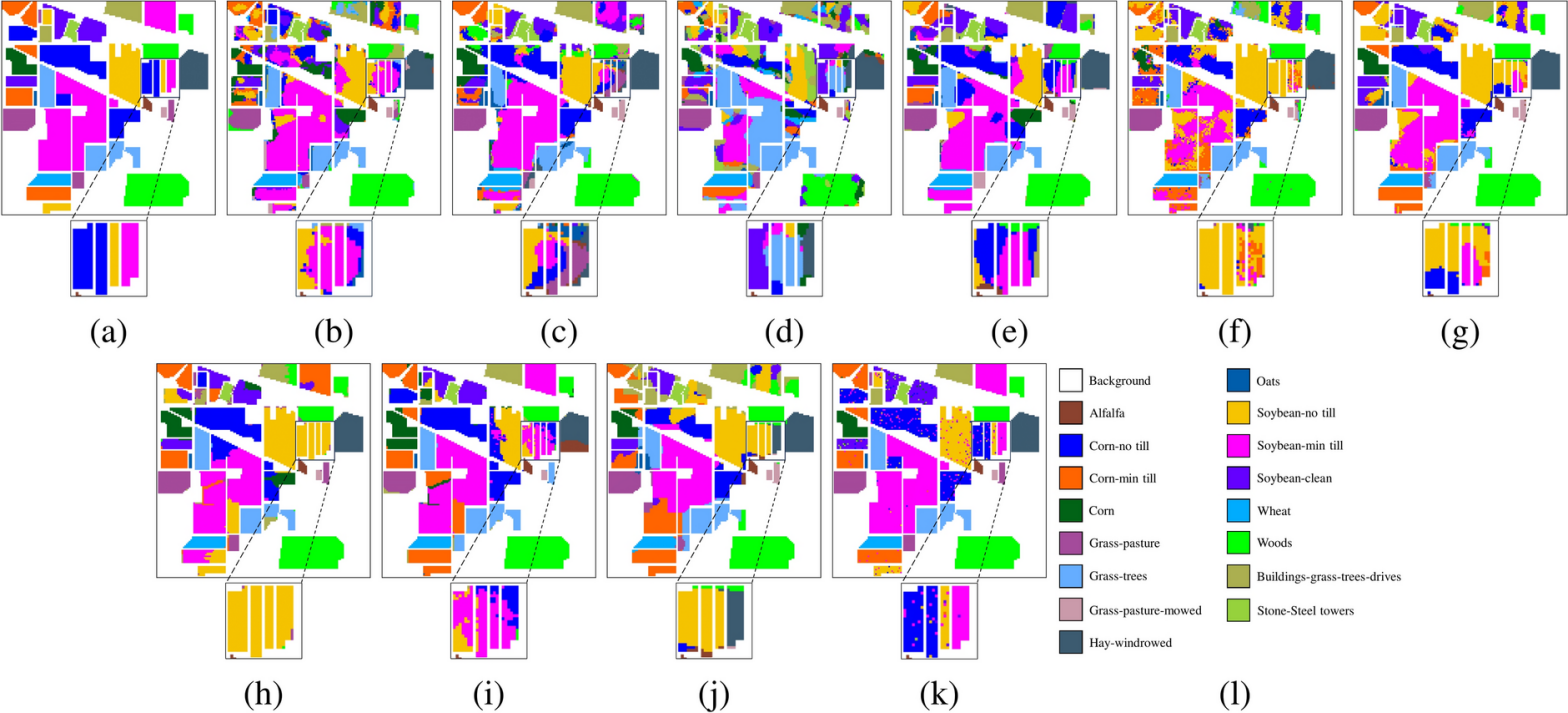
*Indian Pines dataset.* (**a**) Ground truth. Classification maps obtained by different classification methods. (**b**) 2DCNN, OA = 61.76%, (**c**) MCMs, OA = 75.13%, (**d**) HybridSN, OA = 49.72%, (**e**) SSFTT,OA = 70.56%, (**f**) CEGCN,OA = 66.74%, (**g**) MSSGU,OA = 75.89%, (**h**) SBD, OA = 76.95%, (**i**) SHDA, OA = 82.03%, (**j**) AMGCFN, OA = 70.66%, (**k**) MSDFE, OA = 97.85%, (**l**) Labels.

**Table 5:** Classification accuracy (in percent) of different on Salinas dataset with five training samples per class

Class names	2D-CNN	MCMs	HybridSN	SSFTT	CEGCN	MSSGU	SBD	SHDA	AMGCFN	MSDFE
Brocoli-green-weeds-1	97.98(3.04)	**100.00(0.00)**	99.41(1.19)	99.84(0.22)	99.64(0.63)	99.66(0.61)	**100.00(0.00)**	99.84(0.22)	99.57(0.64)	**100.00(0.00)**
Brocoli-green-weeds-2	99.74(0.44)	**100.00(0.00)**	99.86(0.22)	98.85(2.07)	**100.00(0.00)**	99.98(0.03)	99.91(0.10)	98.85(2.07)	99.66(0.46)	**100.00(0.00)**
Fallow	89.48(7.96)	84.71(12.13)	98.25(3.10)	99.84(0.19)	99.70(0.86)	95.43(7.16)	**100.00(0.00)**	99.84(0.19)	99.44(0.99)	99.86(0.20)
Fallow-rough-plow	99.75(0.25)	**100.00(0.00)**	91.72(7.72)	97.67(2.53)	99.48(0.62)	98.89(2.18)	99.93(0.01)	97.67(2.53)	97.65(2.82)	**100.00(0.00)**
Fallow-smooth	91.19(4.13)	97.52(3.60)	93.97(5.55)	94.21(4.19)	96.72(1.77)	96.09(4.17)	99.33(0.00)	94.21(4.19)	92.67(4.07)	**99.99(0.01)**
Stubble	**100.00(0.00)**	**100.00(0.00)**	98.66(1.10)	99.03(1.35)	99.57(0.36)	99.70(0.43)	99.92(0.00)	99.03(1.35)	98.82(1.07)	**100.00(0.00)**
Celery	98.83(1.56)	**100.00(0.00)**	99.09(1.75)	99.78(0.50)	99.54(1.10)	99.33(1.08)	99.44(0.36)	99.78(0.50)	98.41(3.19)	**100.00(0.00)**
Grapes-untrained	58.21(22.94)	74.20(12.72)	72.00(9.61)	73.24(12.80)	71.12(10.70)	80.76(19.52)	78.28(17.94)	73.24(12.80)	77.18(9.80)	**88.65(7.13)**
Soil-vinyard-develop	98.23(2.20)	**100.00(0.00)**	99.03(1.35)	99.92(0.09)	**100.00(0.00)**	99.93(0.17)	99.56(0.66)	99.92(0.09)	**100.00(0.00)**	**100.00(0.00)**
Corn-senesced-green-weeds	83.35(7.51)	97.48(2.38)	96.28(1.66)	92.10(4.44)	90.43(7.15)	87.78(9.29)	95.56(5.86)	92.10(4.44)	94.12(4.25)	**99.92(0.09)**
Lettuce-romaine-4wk	97.42(3.82)	**100.00(0.00)**	98.19(3.27)	99.84(0.23)	99.50(0.46)	99.63(0.51)	97.59(1.20)	99.84(0.23)	96.27(4.25)	**100.00(0.00)**
Lettuce-romaine-5wk	98.80(1.86)	98.68(1.40)	88.03(9.11)	97.30(4.41)	99.88(0.32)	99.42(0.69)	**100.00(0.00)**	97.30(4.41)	99.22(1.76)	**100.00(0.00)**
Lettuce-romaine-6wk	99.36(0.63)	**100.00(0.00)**	97.71(2.91)	97.60(2.06)	99.63(0.81)	96.37(6.43)	98.01(0.63)	97.60(2.06)	98.81(1.63)	**100.00(0.00)**
Lettuce-romaine-7wk	99.29(0.59)	**100.00(0.00)**	92.00(8.06)	93.32(6.80)	98.20(1.62)	96.99(4.70)	93.09(9.46)	93.32(6.80)	98.53(1.63)	**100.00(0.00)**
Vinyard-untrained	76.08(20.55)	99.31(0.65)	81.54(11.62)	87.70(9.04)	92.04(5.44)	86.61(12.01)	88.14(9.99)	87.70(9.04)	86.89(8.08)	**99.82(0.29)**
Vinyard-vertical-trellis	95.53(2.45)	**100.00(0.00)**	90.86(11.49)	95.58(8.36)	98.51(2.49)	86.17(21.56)	97.48(0.76)	95.58(8.36)	98.35(2.90)	**100.00(0.00)**
OA	85.60(2.70)	93.65(4.67)	89.63(1.61)	91.34(2.29)	91.96(2.21)	92.37(3.34)	93.17(3.35)	91.34(2.29)	92.25(2.39)	**97.60(1.48)**
AA	92.70(1.57)	96.99(1.25)	93.57(1.56)	95.37(0.95)	96.50(0.82)	95.17(1.99)	96.64(1.31)	95.37(0.95)	95.97(1.19)	**99.27(0.44)**
$\kappa$	84.07(4.04)	92.98(5.13)	88.52(1.77)	90.40(2.52)	91.10(2.43)	91.52(3.67)	92.43(3.70)	90.40(2.52)	91.40(2.64)	**97.34(1.64)**

The best results are highlighted in bold

**Fig. 9:**
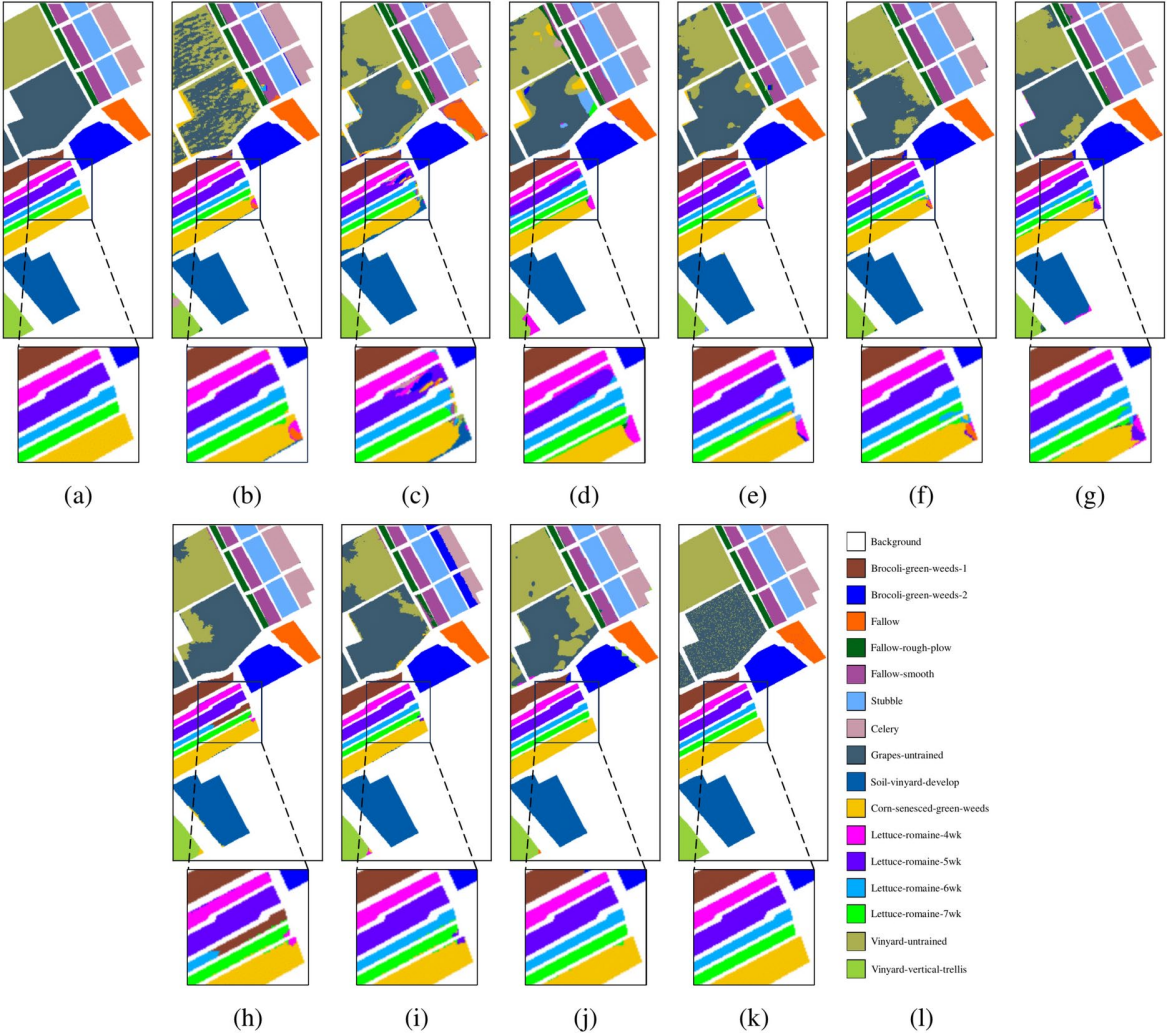
*Salinas dataset.* (**a**) Ground truth. Classification maps obtained by different classification methods. (**b**) 2DCNN, OA = 84.22%, (**c**) MCMs, OA = 90.32%, (**d**) HybridSN, OA = 90.78%, (**e**) SSFTT, OA = 93.99%, (**f**) CEGCN, OA = 92.79%, (**g**) MSSGU, OA = 94.91%, (**h**) SBD, OA = 94.30%, (**i**) SHDA, OA = 92.07%, (**j**) AMGCFN, OA = 92.91%, (**k**) MSDFE, OA = 97.85%, (**l**) Labels.

**Table 6:** Classification accuracy (in percent) of different on Pavia University dataset with five training samples per class

Class names	2D-CNN	MCMs	HybridSN	SSFTT	CEGCN	MSSGU	SBD	SHDA	AMGCFN	MSDFE
Asphalt	66.48(14.04)	18.11(10.45)	30.63(20.54)	65.73(10.14)	90.34(3.56)	81.41(13.38)	63.79(8.71)	78.29(7.09)	73.05(12.95)	**94.67(3.21)**
Meadows	71.67(11.33)	79.33(7.71)	65.93(14.43)	76.12(10.25)	84.49(7.23)	81.91(10.69)	68.59(15.25)	74.63(10.51)	82.12(4.30)	**98.63(1.23)**
Gravel	63.11(11.14)	92.35(4.31)	76.08(17.27)	81.43(7.71)	88.85(12.90)	91.52(7.79)	76.03(19.38)	78.93(9.14)	88.05(8.24)	**98.88(0.74)**
Trees	91.53(7.76)	98.46(1.13)	63.99(12.53)	85.08(6.97)	87.55(7.66)	94.25(3.89)	53.84(6.22)	60.04(7.21)	77.15(9.81)	**99.82(0.14)**
Painted metal sheets	99.97(0.07)	**100.00(0.00)**	99.50(1.08)	99.66(0.78)	99.90(0.29)	99.93(0.20)	96.95(0.02)	97.70(2.71)	99.53(1.09)	**100.00(0.00)**
Bare Soil	63.55(15.61)	85.81(6.56)	55.88(28.03)	80.88(11.61)	97.50(3.12)	93.28(9.54)	88.15(9.01)	86.51(10.23)	96.33(3.09)	**98.85(1.21)**
Bitumen	66.99(24.17)	99.07(0.87)	88.48(22.49)	98.78(1.02)	99.80(0.25)	99.32(2.02)	92.48(2.51)	**100.00(0.00)**	94.67(6.44)	**100.00(0.00)**
Self-Blocking Bricks	52.47(16.11)	53.43(19.12)	38.93(25.68)	52.38(10.33)	92.65(4.35)	88.03(13.55)	81.68(7.21)	81.19(14.35)	96.56(1.74)	**98.28(1.95)**
Shadows	96.39(5.04)	99.88(0.14)	38.03(16.27)	90.70(6.25)	91.41(8.97)	96.94(3.11)	78.25(7.25)	94.96(5.70)	87.34(10.53)	**100.00(0.00)**
OA	70.54(4.95)	72.09(4.42)	58.44(8.47)	75.60(5.38)	89.17(3.73)	86.48(4.39)	72.42(6.73)	78.28(5.28)	84.61(2.06)	**98.23(0.88)**
AA	74.69(3.25)	80.72(2.85)	61.93(7.30)	81.09(2.56)	92.50(3.06)	91.84(1.98)	77.75(2.69)	83.58(2.23)	88.31(2.26)	**98.79(0.57)**
$\kappa$	62.76(5.58)	65.41(4.94)	48.61(9.36)	69.23(6.10)	86.13(4.61)	82.81(5.24)	65.68(7.55)	72.64(6.04)	80.27(2.58)	**97.65(1.16)**

The best results are highlighted in bold

**Fig. 10:**
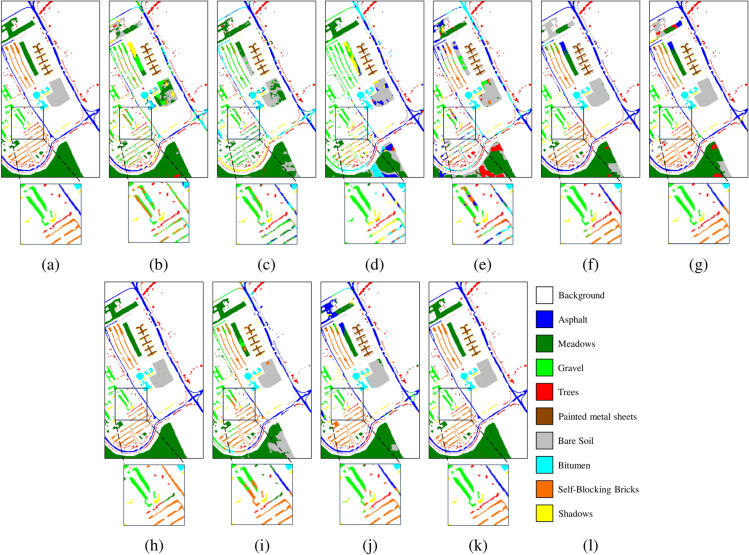
Pavia University dataset. (**a**) Ground truth. Classification maps obtained by different classification methods. (**b**) 2DCNN, OA = 67.83%, (**c**) MCMs, OA = 70.30%, (**d**) HybridSN, OA = 62.08%, (**e**) SSFTT, OA = 63.50%, (**f**) CEGCN, OA = 84.97%, (**g**) MSSGU, OA = 83.96%, (**h**) SBD, OA = 78.46%, (**i**) SHDA, OA = 77.48%, (**j**) AMGCFN, OA = 85.46%, (**k**) MSDFE, OA = 97.85%, (**l**) Labels.

**Fig. 11:**
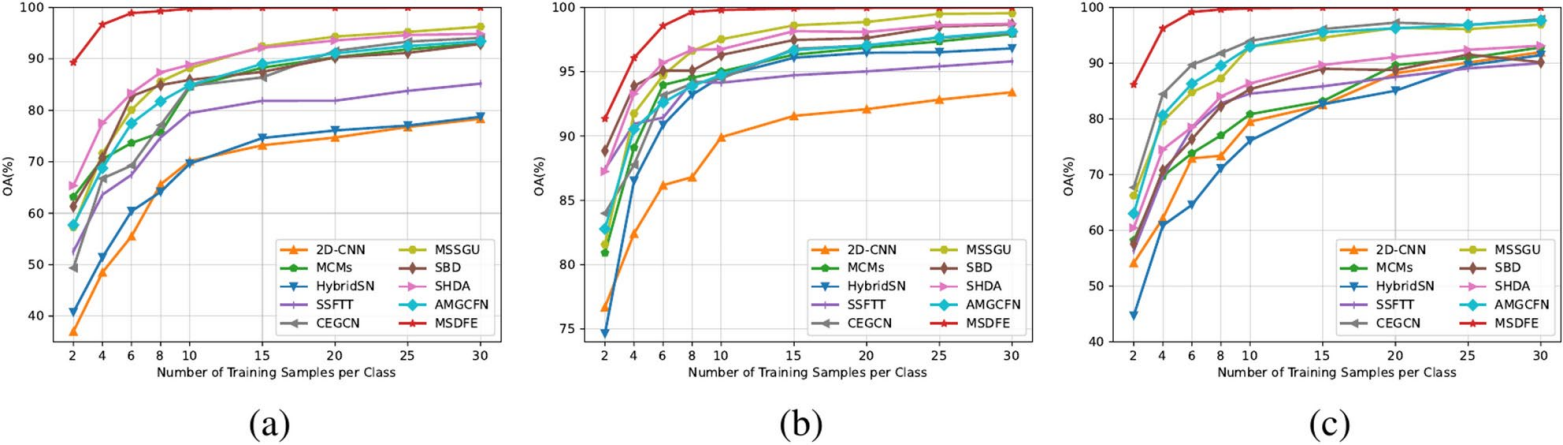
Effect of the number of training samples on the 2D-CNN, MSDFE, HybridSN, SSFTT, CEGCN, MSSGU, SBD, SHDA, AMGCFN, and MSDFE methods for (**a**) Indian Pines dataset, (**b**) Salinas dataset, and (**c**) Pavia University dataset.

The quantitative results on the three datasets are listed in Tables 4, 5, and 6, with the best results highlighted in bold. The corresponding classification maps are provided in Figs. 8, 9, and 10. The results indicate that the MSDFE method exhibits outstanding classification accuracy and robustness, especially under conditions with complex categories and limited training samples.

For the Indian Pines dataset, the OA of the proposed method is 98.12%, which is 43.98% higher than the worst-performing method, HybridSN, and 16.17% higher than the best-performing method, SHDA. For the categories with highly similar spectral features but localized differences in spatial distribution, such as Corn-notill and Corn-mintill, the proposed method achieves high accuracies of 92.29% and 98.69%, respectively, which are much higher than other comparison methods. This high accuracy is mainly attributed to the MSDFE method, which aggregates spatially adjacent and spectrally similar pixels into regions through superpixel segmentation, thereby reducing the impact of spectral aliasing.

For the Salinas dataset, the proposed method shows an OA of 97.40%, along with an AA of 99.27% and a $\kappa$ coefficient of 0.9734. These results surpass the best-performing method, MCMs, by 3.95%, 2.28%, and 4.36%, respectively. Due to the uniform distribution of categories and the obvious spectral differences between categories in the Salinas dataset, most methods achieve good classification accuracy. However, the proposed MSDFE method still achieves the best performance because it effectively utilizes multi-scale superpixel features to capture complex boundary and local details, improving classification accuracy.

For the Pavia University dataset, methods that combine superpixels and deep learning, such as CEGCN, MSSGU, and AMGCFN, perform well, achieving a maximum OA of 89.17%. However, methods solely relying on CNN or superpixels perform poorly, with a maximum OA of 78.28%. In contrast, the proposed method demonstrates significant superiority on this dataset, achieving an OA of 98.23%, which surpasses the best result from the aforementioned methods by 9.06%. This may be attributed to the fact that the proposed method puts different scales of superpixels through a deep learning module and performs adaptive fusion to more accurately capture the complex spatial and spectral information of HSI.

The improvement in classification performance is also reflected in the classification maps generated by the proposed method, and some hard-to-distinguish regions are enlarged to display the details of the classification results. From these results, it can be seen that for the Indian Pines and Pavia University datasets, boundaries of complex categories are more clearly defined, with fewer misclassified regions. For the Salinas dataset, the boundary transitions between categories are smooth. Particularly for complex classes such as Lettuce-romaine, the proposed method accurately identifies subtle variations, further validating its remarkable enhancement of classification accuracy in HSI.

To further validate the proposed MSDFE method, we have also investigated the influence of the number of training sample for the compared methods on three datasets. In experiments, we randomly select different numbers of sample from each class to serve as the training set. The number of the selected labeled samples per class is set from 2 to 30. Specifically, the step size is 2 in the range from 2 to 10 and the step size is 5 in the range from 10 to 30, respectively. Considering that the labeled sample number of some categories for the Indian Pines dataset (Alfalfa, Grass-pasture-mowed, Oats) is less than 50, half of the labeled samples are selected as the training samples when the number of training sample is over half of the total number of samples in these categories. As shown in Fig. 11, with the number of training sample increases, the performance for all the considered HSI classification methods are generally improved. Most importantly, the classification performance of the proposed MSDFE method consistently outperforms all comparison methods. Although the number of training sample is small, the proposed MSDFE method shows significant advantage in classification performance. These results further evidence that the proposed MSDFE method, by constructing covariance matrices and utilizing deep learning network to fuse spatial and spectral information coming from different scale superpixels, can obtain features with more discriminative capability for HSI classification.

## Conclusion

In this article, a novel MSDFE method has been proposed for HSI classification. In this method, by constructing two-dimensional statistical features, the spatial-spectral features contained within the superpixel block can be naturally fused and effectively extracted. Moreover, the statistical features extracted from superpixel blocks of different shapes share the same size, which facilitates further learning depth features through a unified CNN model. In addition, the complex structure of ground objects in HSI makes single scale superpixel segmentation prone to over-segmentation or under-segmentation, while multiscale segmentation can effectively address these issues. Therefore, in the proposed method, the multiscale superpixel segmentation is used to capture the information of different scales and effectively fuse, further enhancing the classification accuracy. Experiments on three real-world HSI datasets show that the proposed MSDFE method outperforms the existing classical and state-of-the-art HSI classification methods, especially in terms of classification performance under small sample conditions. In future work, we consider fusing pixel-level features with multiscale superpixel-level features to construct a more discriminative feature, which will improve classification performance. Moreover, we will also adaptively select fusion scales to accommodate different datasets, which can better balance the computational cost and classification performance of the algorithm.

## Data Availability

The datasets analyzed during the current study are available in https://www.ehu.eus/ccwintco/index.php?title=Hyperspectral_Remote_Sensing_Scenes.
